# *Streptococcus gallolyticus* and Bacterial Endocarditis in Swine, United States, 2015–2020 

**DOI:** 10.3201/eid2801.210998

**Published:** 2022-01

**Authors:** Panchan Sitthicharoenchai, Eric R. Burrough, Bailey L. Arruda, Orhan Sahin, Jessica G. dos Santos, Drew R. Magstadt, Pablo E. Piñeyro, Kent J. Schwartz, Michael C. Rahe

**Affiliations:** Iowa State University, Ames, Iowa, USA (P. Sitthicharoenchai, E.R. Burrough, O. Sahin, J.G. dos Santos, D.R. Magstadt, P.E. Piñeyro, K.J. Schwartz, M.C. Rahe);; US Department of Agriculture, Ames (B.L. Arruda)

**Keywords:** Streptococcus gallolyticus, bacterial endocarditis, swine, bacteria, United States, Streptococcus bovis

## Abstract

To evaluate trends in bacterial causes of valvular endocarditis in swine, we retrospectively analyzed 321 cases diagnosed at Iowa State University Veterinary Diagnostic Laboratory (Ames, IA, USA) during May 2015–­April 2020. *Streptococcus gallolyticus* was the causative agent for 7.59% of cases. This emerging infection in swine could aid study of endocarditis in humans.

Bacterial endocarditis appears as the distinctive macroscopic lesions of vegetative valvular endocarditis (VVE). Among humans, one of the main causes of infective endocarditis is *Streptococcus gallolyticus* (formerly *Streptococcus bovis*), which reportedly causes 2%–10% of cases (*1*). Despite a lack of reports of *S. gallolyticus* pathogenicity in swine, this bacterium is considered to be part of the porcine enteric microbiome (*2*). The classic causes of swine VVE include *Erysipelothrix rhusiopathiae*, *Streptococcus suis*, and *Trueperella pyogenes* (*3**,**4*); however, knowledge of bacteria associated with VVE in swine is limited. To evaluate trends in bacteria isolated from swine with VVE, we retrospectively analyzed cases submitted to the Iowa State University Veterinary Diagnostic Laboratory (ISU VDL; Ames, IA, USA) during May 2015–April 2020. 

## The Study

During the 5-year period, ISU VDL diagnosed 321 cases of swine VVE in pigs 3–28 weeks of age. Cases were submitted from 20 states, including major swine-producing states in the US Midwest. For 255 (79.43%) of these cases, the causative agent(s) were detected by routine bacterial culture of the affected heart valves. Heart valves were swabbed with sterile cotton swabs and plated onto 5% sheep blood agar plates, and plates were incubated in 5% CO_2_ at 35°C and examined after 18–24 h and 48 h. Broth enrichment for *Erysipelothrix* spp. (*5*,*6*) was performed according to the discretion of the diagnostician. Bacterial identification was based on colony morphology, followed by speciation based on matrix-assisted laser desorption/ionization time-of-flight mass spectrometry (Bruker Daltonix, https://www.bruker.com). 

For this study, we searched ISU VDL pathology reports for May 2015–April 2020 to identify all cases of endocarditis in swine based on the gross changes of vegetative endocarditis. For selected cases from which pure growth of *S. gallolyticus* was isolated from the heart valves, we performed bacterial biochemical analyses, sequenced 16S ribosomal RNA, and localized *S. gallolyticus* within lesions via RNA in situ hybridization (ISH) (RNAscope; TriStar Technology LLC, https://tristargroup.us). We also used immunohistochemistry to identify underlying immunosuppression. 

We recovered a total of 290 bacterial isolates from the VVE lesions: *S. suis* (196, 67.59%), *S. equisimilis* (37, 12.76%), *S. gallolyticus* (22, 7.59%), other *Streptococcus* spp. (10, 3.45%), *E. rhusiopathiae* (9, 3.10%), *Actinobacillus* spp. (9, 3.10%), *T. pyogenes* (3, 1.04%), *Enterococcus faecalis* (2, 0.69%), *Vagococcus fluvialis* (1, 0.34%), and *Staphylococcus aureus* (1, 0.34%) (Table). Single bacterial pathogens were isolated from 221 of the 321 swine with VVE; multiple bacterial pathogens were isolated from 34. Of the remaining 66 swine, no confirmed cause of VVE was determined because of lack of heart valve submission for bacterial culture (47, 71.21%), bacterial contamination (15, 22.73%), or lack of bacterial growth (4, 6.06%). 

**Table:** Frequency of bacterial pathogen isolation from 255 swine with determined causes of bacterial endocarditis, Iowa State University Veterinary Diagnostic Laboratory, Ames, Iowa, USA, 2015–2020

Bacterial pathogens	Frequency of isolation, no. (%)	Sole bacterium isolated, no. cases	Isolated in mixed infection, no. cases
*Streptococcus* spp.			
* S. suis*	196 (67.59)	166	30
* S. equisimilis*	37 (12.76)	15	22
* S. gallolyticus*	22 (7.59)	21	1
Other streptococci	10 (3.45)	7	3
*Erysipelothrix rhusiopathiae*	9 (3.10)	5	4
*Actinobacillus* spp.	9 (3.10)	2	7
*Trueperella pyogenes*	3 (1.04)	1	2
*Enterococcus faecium*	2 (0.69)	2	0
*Vagococcus fluvialis*	1 (0.34)	1	0
*Staphylococcus aureus*	1 (0.34)	1	0
Total bacteria*	290 (100)	Not applicable	Not applicable

*Calculations based on 290 isolations from 255 cases.

## Conclusions

**Figure 1:**
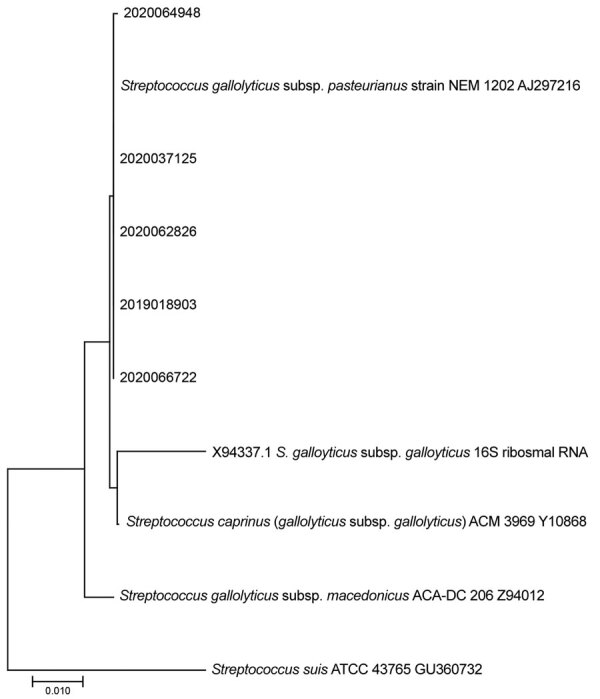
Five selected *Streptococcus gallolyticus* isolates from swine vegetative valvular lesions (2019018903, 2020037125, 2020062826, 2020064948, and 2020066722) characterized as *S. gallolyticus* subspecies *pasteurianus* on the basis of reference 16S ribosomal RNA sequences. Samples were collected in the United States during 2015–2020. Scale bar indicates nucleotide substitutions/site.

**Figure 2:**
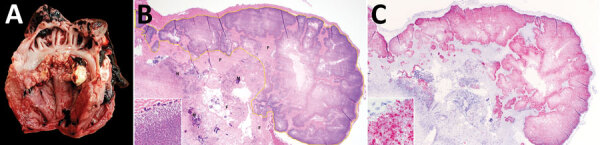
Lesions associated with swine vegetative endocarditis, United States, 2015–2020. A) Macroscopic findings of vegetative growth on the left atrioventricular heart valve leaflets. B) Histopathologic findings of inflammation characterized by necrotic leukocytes (N), fibrin (F), mineralization (M), and myriad bacterial colonization (yellow outline) along the surface of the heart valve (hematoxylin and eosin staining); original magnification ×40. Higher magnification image (inset) shows cocci bacteria in clusters and long chains; original magnification ×1,000. C) *Streptococcus gallolyticus* directly detected (red) on the surface of the heart valve by RNA in situ hybridization with a probe targeting the helix-hairpin helix domain–containing protein, ComEC/Rec2, and DNA pol III subunit delta genes specific to *S. gallolyticus*; original magnification ×40. Higher magnification image (inset) shows the bacteria labeled by the in situ hybridization probe; original magnification ×1,000.

Within our dataset, the relatively high proportion of swine VVE cases associated with *S. gallolyticus­* compared with other recognized VVE-associated pathogens in swine (e.g., *E. rhusiopathiae* and *T. pyogenes*) was unexpected. *S. gallolyticus* is part of Lancefield group D streptococci with subclassification of *S. gallolyticus* subspecies *gallolyticus* (formerly *S. bovis* biotype I) and *S. gallolyticus* subsp. *pasteurianus* (formerly *S. bovis* biotype II/2) (*7*). This pathogen has been linked to 2%–10% of infective endocarditis cases in humans (*1*) as well as to human colon cancer (*8*,*9*). Lancefield group D streptococci have been described as commensal flora in the gastrointestinal tract of birds and mammals, including swine (*2*). Bescucci et al. (*10*) identified *S. gallolyticus* within areas of enteric inflammation in pigs challenged with *Salmonella enterica* serovar Typhimurium. That finding suggests that injury to intestinal mucosa might predispose pigs to septicemia and subsequent formation of *S. gallolyticus*–associated VVE. According to our 16S ribosomal RNA sequencing of 5 selected *S. gallolyticus* isolates from VVE-infected swine, all isolates were classified as *S. gallolyticus* subsp. *pasteurianus* (Figure 1). Biochemical testing indicated that the isolates were positive for trehalose and esculin and weakly positive for inulin and mannitol. The causative association of *S. gallolyticus* isolated from VVE lesions was confirmed by ISH (Figure 2).

Predisposing causes of VVE development in swine are unknown; however, porcine reproductive and respiratory virus (PRRSV) and porcine circovirus 2 (PCV2) are notable immunosuppressive viruses that might increase susceptibility of a pig to bacterial infection and septicemia. Underlying PRRSV and PCV2 infection was determined for 17 of 22 swine with *S. gallolyticus–*associated VVE by using available formalin-fixed paraffin-embedded tissue blocks and immunohistochemistry staining of lung tissue, lymphoid tissue, or both. Only 2 of 17 swine were immunopositive for PRRSV, and all were negative for PCV2. This finding suggests that common immunosuppressive viral infections are not required for development of *S. gallolyticus* infection. Another compelling predisposing factor that warrants further investigation is intestinal mucosal damage in association with formation of *S. gallolyticus* porcine VVE; however, the temporal association between these events must be considered.

The pathogenesis of VVE is not completely known. Factors involved with development of lesions include the presence of transient/persistent bacteremia and preexisting damage of the valvular surface exposed to blood flow (*1*). Streptococci are commonly associated with VVE formation across species. Expression of microbial surface components (e.g., fibrinogen/fibronectin binding protein, collagen binding protein, and pili) that recognize host extracellular matrix molecules and increase adherence to damaged heart valves have been identified in various species of streptococci, including *S. gallolyticus* (*11*). Furthermore, streptococcal capsular protein has been shown to inhibit complement formation, leading to bacterial survival in the cardiovascular system (*12*).

Distribution of bacterial pathogens associated with swine VVE varies notably by age of the pigs. Age range for detection of *S. suis* was the widest, 3–24 weeks, and incidence peaked at 12–15 weeks. Similarly, *S. equisimilis* was detected in pigs 6–28 weeks of age and *S. gallolyticus* at 6–19 weeks of age; *S. gallolyticus* incidence peaked in 12-week-old pigs. Our data indicated a low number of *E. rhusiopathiae* isolations from VVE lesions, in contrast to previous reports of VVE in slaughter-age pigs (*2*). One major contributing factor to that finding is implementation of swine erysipelas vaccination of breeding herds, providing passive immunity and protection in nursery pigs (*13*), leading to a higher tendency to isolate this bacterium in the pigs at the grower/finisher stage of development (>12 weeks). However, inconsistent inclusion of *Erysipelothrix* spp. enrichment culture could have limited detection of this pathogen in our dataset.

Epidemiologic data for *S. gallolyticus* distribution in swine herds is limited. The reported *S. gallolyticus*–associated VVE cases were from different locations in 6 states (Missouri [5/22], Iowa [4/22], Illinois [3/22], Indiana [3/22], Arkansas [2/22], North Carolina [2/22]); 3 cases were from undetermined locations. Information regarding the type of swine production system and genetic sources was not recorded. In addition, this geographic information is limited to cases that were submitted to the ISU VDL. Thus, association with predisposing factors, such as husbandry and management, and the endemic status of this pathogen among domestic swine is unknown.

Our data on the frequency of detection of bacterial agents in swine with VVE reshapes the contemporary understanding of common causes of VVE in US domestic swine herds and identifies *S. gallolyticus* as an emerging cause of bacterial endocarditis in swine. This finding is supported by the frequent isolation of pure-growth *S. gallolyticus* from affected valves and direct detection of this agent within lesions via ISH. Determining factors that predispose swine to systemic infection, examining lesion distribution in other tissues, and reproducing the disease in an experimental setting would position swine as a highly translatable model of *S. gallolyticus* infectious endocarditis for the study and treatment of endocarditis in humans.
